# The Germ Theory Controversy

**Published:** 1877-09

**Authors:** 


### The Germ Theory Controversy.

In our last we gave a *resume* of Professor Tyndall's experiments and his lecture on the subject at the Royal Institution. Dr. Charlton Bastian has since addressed a letter to one of the daily papers, in which he questions the soundness of Tyndall's reasoning in his latest experiments on germs.

"In January of the present year," writes Dr. Bastain, "and now again in June, we are told by Professor Tyndall, that the conclusions drawn from his former experiments were hasty and erroneous. We are assured:—1. That guarded infusions, previously boiled, even for several hours, will frequently become corrupt and putrid. 2. That the germs of ferment organisms, instead of being uniformly killed by five minutes' boiling, will often resist the destructive process for several hours. But not at all discomfited, Professor Tyndall still bids his audienee believe that there is not 'a shadow of evidence in favour of spontaneous generation.'

"I would in the first place point out," says Dr. Bastain, "that the great divergence as to fact between Professor Tyndall and other investigators, to which I was permitted to call the attention of your readers in January, 1876, now no longer exists. He is at one with us as to the frequent fertility of boiled infusions, so that the only question between us, at present, is one of interpretation. This, as I freely admitted, is a subject for discussion, on which there is room for some difference of opinion. I am far from wishing to parapharse Professor Tyndall's expression and say there is not a shadow of evidence in favour of the germ theory, though I do strongly believe that it never can be supported on such partial evidence and arguments as those to which Professor Tyndall has had recourse.

"There are two important questions of fact amid many others, underlying the attempt now being made by Professor Tyndall to reconcile his earlier with his later experiments. They are these: —1. Have Bacteria and their germs or not a power of resisting prolonged dessication? 2. At what precise temperature are Bacteria and their germs killed when immersed in fluids? Professor Tyndall has never made known any decisive evidence bearing upon either of these questions. The former has, however, been made the subject of investigation by Professor Burdon Sanderson, and the latter by myself. Neither of us committed the grave error of 'confounding the germ with the offspring.' Any of your readers may see in the 'Thirteenth Report of the Medical Officer of the Privy Council,' p. 61, that Professor Sanderson saw reason for concluding that both Bacteria and Bacteria germs were unable to resist the dessicating influence even of a temperature so low as 104 deg. Fahrenheit for two or three days; while the evidence which I adduced in 1874, to show that Bacteria were killed at about the temperature of 140 deg. Fahrenhhit showed, as I then pointed out, just as conclusively that this temperature was destructive to Bacteria germs—that is, to all the reproductive particles, whether visable or invisable, by which these organisms multiply in suitable fluids.

"Much of the evidence by which Professor Tyndall seeks to support his present position, has no real cogency, because, while following, as he says, 'the plain indications of the germ theory,' he is, unfortunately, oblivious of the equally plain indications of the opposite theory. Results which can be equally well explained in accordance with two conflicting views, can never have any decisive value in establishing one of them.—*London Medical Press.*

